# The three-decade trajectory of hepatitis C burden among women of reproductive age in China: a retrospective and predictive study

**DOI:** 10.1186/s12985-026-03079-4

**Published:** 2026-05-21

**Authors:** Ying Zhang, Jing Yan, Meng Li, Chaoyan Yue

**Affiliations:** 1 Shanghai Key Lab of Reproduction and Development, Shanghai Key Lab of Female Reproductive Endocrine Related Diseases, Obstetrics & Gynecology Hospital of Fudan University, Shanghai, China; 2 Department of Laboratory Medicine, Jinshan Hospital of Fudan University, Shanghai, China

**Keywords:** Hepatitis C Virus (HCV), Disease burden, Epidemiological trends, Age-period-cohort analysis, Global Burden of Disease (GBD)

## Abstract

**Background:**

This study evaluates the changing burden of hepatitis C (HCV) among women of reproductive age (15–49 years) in China from 1990–2021 and forecasts trends to 2035.

**Methods:**

Using data from the Global Burden of Disease 2021 study, we analyzed age-standardized rates (ASRs) for incidence, prevalence, mortality, and disability-adjusted life years (DALYs) related to acute and chronic HCV in this population. Statistical analyses included estimating annual percentage changes (EAPCs), applying Poisson-based age-period-cohort (APC) modeling to assess epidemiological drivers, and employing Bayesian APC modeling for future projections. Decomposition analysis was used to quantify contributions from population growth, aging, and epidemiological factors.

**Results:**

From 1990 to 2021, all ASRs for HCV among women of reproductive age in China showed significant declines, with rates of decrease surpassing global averages. Despite this progress, China remained a high-burden country for this group in 2021, with a large reservoir of chronic HCV cases. A notable rebound in the incidence of chronic HCV occurred after 2010. Epidemiological trends displayed clear age stratification: the most substantial improvements were observed in younger cohorts (aged 15–24 years), while older groups (aged 45–49 years) experienced slower declines or recent rebounds in incidence. Decomposition analysis indicated that the reduction in disease burden was primarily driven by epidemiological improvements (e.g., in prevention and treatment), which successfully offset the adverse effects of population growth. This pattern contrasts with the global trend, where increasing burden is largely attributed to demographic factors. Projections suggest a continued decline in ASRs and case numbers through 2035, although the rate of decline is expected to slow.

**Conclusions:**

Historical HCV control measures for women of reproductive age in China have been effective, as evidenced by steep declines in mortality and DALYs. However, persistent challenges include a substantial existing patient pool and a resurgent incidence, particularly among older individuals within this demographic. This study precisely quantifies a successful epidemiological transition and identifies a shift in risk profiles, underscoring the continued need for sustained, age-tailored public health interventions to achieve HCV elimination goals.

**Supplementary Information:**

The online version contains supplementary material available at 10.1186/s12985-026-03079-4.

## Background

Hepatitis C virus (HCV) infection continues to pose a significant global public health burden [1], according to data from the World Health Organization’s 2022 Global Hepatitis Report, approximately 58 million people worldwide are living with chronic hepatitis C virus infection. [2, 3], representing a leading cause of liver cirrhosis, hepatocellular carcinoma (HCC), and liver-related mortality [4, 5]. While the advent of highly effective direct-acting antiviral (DAA) therapies has revolutionized treatment paradigms [6–8], substantial challenges remain in achieving the World Health Organization’s (WHO) 2030 elimination targets of 90% reduction in new infections and 65% decrease in mortality, particularly in low- and middle-income countries (LMICs) where constrained healthcare resources, limited screening programs, and diagnostic bottlenecks persist [9–12]. The natural history of HCV infection highlights the critical importance of the acute phase, where despite spontaneous clearance occurring in 30–50% of cases [13, 14], the majority progress to chronic infection characterized by persistent viremia beyond six months, creating an imperative for early detection and intervention to prevent long-term hepatic complications [1, 15], interrupt transmission chains, particularly among high-risk populations, and optimize clinical outcomes through timely linkage to care.

The Global Burden of Disease (GBD) study provides comprehensive estimates of HCV-related health metrics, enabling cross-country comparisons and trend analyses that are critical for understanding the global epidemiology of hepatitis C [16, 17]. While GBD data have been widely used to assess HCV burden worldwide, including analyses of incidence, mortality, and disability-adjusted life years (DALYs), few studies have specifically examined China’s unique HCV dynamics in depth regarding women of reproductive age, particularly the distinct patterns of acute and chronic infection across different regions and age groups [18, 19].According to the latest epidemiological estimates released by the Chinese Center for Disease Control and Prevention in 2021, China faces a substantial burden of hepatitis C, with approximately 10 million chronic hepatitis C cases and a general population prevalence of about 0.91% [20, 21].—existing GBD-based analyses often lack detailed stratification by disease stage (acute vs. chronic) and specific population groups such as women of reproductive age or fail to incorporate advanced modeling techniques to disentangle the effects of aging, temporal interventions, and birth cohort risks [22]. This study was conducted within the context of China’s "Action Plan for Eliminating the Public Health Threat of Hepatitis C (2021–2030)" [23], which outlines the national strategy to address this substantial disease burden.which sets ambitious targets, including a 90% reduction in new infections and 80% treatment coverage by 2030. The findings presented here on the evolving epidemiology of HCV among women of reproductive age in China are intended to provide an evidence base for monitoring progress toward these goals.

To address these gaps, we conducted a comprehensive analysis of acute and chronic HCV burden among women of reproductive age in China from 1990 to 2021 using GBD 2021 data, employing average annual percent change regression to identify temporal trends, age-period-cohort (APC) modeling to assess demographic and temporal influences, and Bayesian forecasting to project disease trajectories through 2035. Additionally, decomposition analysis was applied to quantify the contributions of epidemiological changes, population growth, and aging to HCV burden in this population. This study provides critical insights into HCV transmission dynamics and outcomes among women of reproductive age in China, offering evidence to optimize screening, treatment strategies, and the country’s progress toward HCV elimination.

## Methods

### Data sources

The data of this study are derived from the global burden of disease study 2021 (GBD2021). The GBD database was led and developed by the Institute for Health Metrics and Evaluation (IHME) at the University of Washington in the United States. It includes 371 diseases and injuries from 204 countries and regions around the world.

The resulting disease burden situation aims to systematically quantify the global disease burden, risk factors and health trends, providing data support for policy-making and medical research. Among them, the data of China mainly come from the national population census, the disease surveillance point system and the literature review analysis of the prevalence of related diseases. Based on data from the Global Burden of Disease Study 2021 (GBD 2021), spanning 1990 to 2021, this study extracted epidemiological metrics for acute and chronic hepatitis C among women of reproductive age in China. Key indicators included age-standardized incidence, prevalence, and mortality rates [16, 17].

### Overview of study methods and data sources

This study is a retrospective time-series analysis that utilized data from the Global Burden of Disease Study 2021 (GBD 2021). The GBD estimates for hepatitis C among women of reproductive age in China integrate multiple input sources, including national surveillance data from the China National Notifiable Disease Reporting System (NNDRS) and national population census data.The study population includes all reported hepatitis C cases among women of reproductive age across mainland China from 1990 to 2021. Case definitions strictly adhere to the Diagnostic Criteria for Hepatitis C issued by the National Health Commission of China: an acute hepatitis C case is defined as a newly confirmed individual positive for both anti-HCV and HCV RNA with documented clinical onset within a 6-month period, whereas a chronic hepatitis C case is defined as either a confirmed HCV infection lasting more than 6 months or a newly reported case assessed by the attending physician as not meeting the acute criteria based on clinical and laboratory evaluation. These definitions align in principle with WHO guidelines while reflecting operational distinctions in determining chronicity within China’s surveillance framework. Regarding the surveillance and reporting process, all cases diagnosed at healthcare facilities are mandatorily reported within a specified timeframe through the NNDRS. Initially reported as clinically diagnosed, cases are subsequently verified and classified (acute vs. chronic) by local Center for Disease Control and Prevention staff based on follow-up and supplementary laboratory data. The analysis in this study utilizes the finalized, deduplicated case records for women of reproductive age from this system.

### Statistical analysis

This study conducted a statistical analysis of the disease burden of acute and chronic hepatitis C among women of reproductive age in China using the R language (version 4.4.1). Through the age-standardized rate (ASR) and the estimated annual percentage change EAPC quantifies the changing trends of the incidence rate, prevalence rate, mortality rate and disdisability adjusted life years (DALYs) of acute and chronic hepatitis C in this population from 1990 to 2021. The age-period-cohort (APC) model is based on the Poisson distribution to analyze and explore the influence of age structure, epidemiology and demographic factors on the burden of disease [24–26].The Bayesian age-period-cohort model (BAPC) was constructed in combination with the R package to predict the overall incidence, prevalence, mortality and DALYs of acute and chronic hepatitis C among women of reproductive age in China from 2020 to 2035 [27]. Meanwhile, based on the Global Burden of Disease (GBD) data, the regional and age composition of the incidence and mortality rates of patients in this population was analyzed. Furthermore, the contribution degrees of Population, Aging and Epidemiological changes to the changes in the disease burden of acute and chronic hepatitis C (such as incidence rate, mortality rate, DALYs, etc.) among women of reproductive age in China were quantified by using decomposition analysis. Statistical analyses included descriptive measures reported as means with 95% uncertainty intervals (UIs), and p-values below 0.05 were deemed statistically significant in trend evaluations.

## Results

### The burden of hepatitis C disease among women of reproductive age in China in 2021 and its changing trend from 1990 to 2021

From 1990 to 2021, both the age-standardized incidence rate (ASIR) and prevalence rate (ASPR) of hepatitis C among women of reproductive age in China showed a significant downward trend. Among them, the standardized incidence rate of acute hepatitis C in this population in 2021 was 16.647/100,000, the standardized incidence rate of chronic hepatitis C was 10.651 per 100,000 population, with an EAPC of –2.557% (95% CI: –3.033 to –2.078), and the decline rates were all faster than the global average level (acute EAPC = -0.164; Chronic EAPC = -0.273. In 2021, the number of patients with chronic hepatitis C among women of reproductive age in China was approximately 4.235 million, with an age-standardized prevalence rate of 1,301.019 per 100,000, accounting for 12.5% of the global total cases. This suggests that China remains one of the countries with a high burden of hepatitis C for this group, but the prevention and control achievements are significant (EAPC = -2.727, 95%CI (-3.285, -2.164)). In terms of mortality rate, the number of deaths related to chronic hepatitis C among women of reproductive age in China in 2021 was 476 cases. The age-standardized mortality rate (ASDR) was 0.118/100,000, EAPC = -5.425, 95%CI (-5.575, -5.274), which was significantly lower than the global level (1.417/100,000). And the decline was more obvious (global EAPC = -0.984). The death cases of acute hepatitis C were extremely rare (ASDR = 0.001 per 100,000), but the average annual decline rate EAPC was-14.306, 95%CI (-15.090, -13.515). The Burden of disease (DALY) analysis showed that the overall DALY of hepatitis C among women of reproductive age in China in 2021 was 23,700. The proportion of chronic hepatitis C exceeded 99%, and its standardized DALY rate was 6.005 per 100,000. EAPC = -5.317, 95%CI (-5.465, -5.168). The global figure is 74.071 per 100,000 (EAPC = -0.931). The rate of decline in the standardized incidence rate, mortality rate and DALY rate of hepatitis C among women of reproductive age in China is much higher than that globally, but the scale of the existing patients with chronic hepatitis C in this group is still large. (Table 1).

**Table 1:**
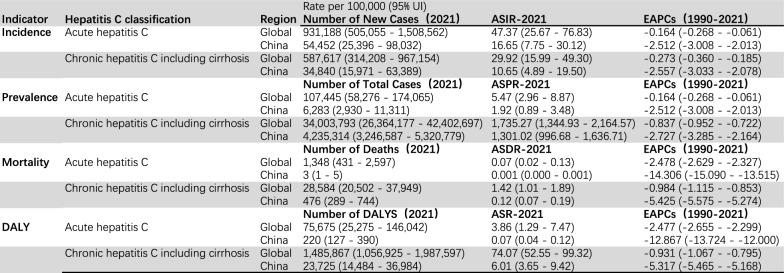
Burden of hepatitis C among women of reproductive age in China and globally in 2021 and temporal trends from 1990 to 2021

### Age-specific burden of acute and chronic hepatitis C among women of reproductive age in China, 1990–2021

**Fig. 1:**
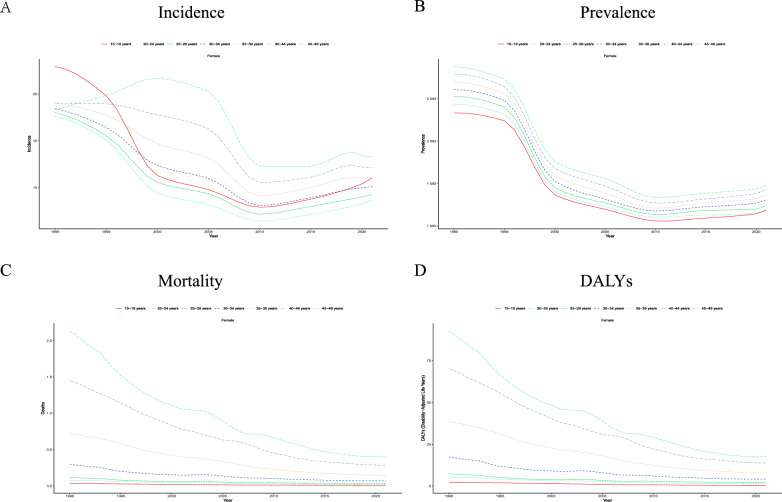
Trends in chronic hepatitis C disease burden among women of reproductive age (15–49 years) in China, by age group, 1990–2020.(**A**) Incidence. (**B**) Prevalence. (**C**) Mortality. (**D**) Disability-adjusted life years (DALYs).Age groups: 15–19 years (solid red line), 20–24 years (dashed orange line), 25–29 years (dotted yellow line), 30–34 years (dash-dotted green line), 35–39 years (long-dashed cyan line), 40–44 years (dash-dot-dotted blue line), and 45–49 years (triple-dotted purple line)

Analysis of the epidemiological trend of chronic hepatitis C among women of reproductive age in China from 2021shows significant age-stratified characteristics: The incidence rates in each age group showed a dynamic change of first decreasing and then increasing. The incidence rate in the 15–19 age group peaked in 1990 and continued to decline to the lowest point in 2010 (with a decrease of approximately 60%). The 20–24 age group peaked around 2000 and then gradually decreased, while the population over 30 years old showed a continuous downward trend but a small rebound in recent years. The analysis of the prevalence rate during the same period showed that all age groups decreased significantly (with an overall decrease of more than 50%), among which the decrease was the largest in the 15–19 age group and the smallest in the 45–49 age group. The changes in mortality rate showed age gradient differences. The mortality rate of the 45–49 age group was the highest between 1990 and 2000, but the subsequent improvement was the most significant, while the 15–19 age group remained at the lowest level all along. DALYs analysis further verified the age-dependent disease burden. Although the initial burden was the heaviest in the 45–49 age group, the decrease was the greatest (p < 0.01). The burden was the lightest and most stable in the 15–19 age group, and the intermediate age group (20–44 years old) showed a continuous improvement trend (Fig 1).

Epidemiological analysis of acute hepatitis C in Chinese women of reproductive age from 1990 to 2021 shows that: The incidence rates of all age groups showed a U-shaped change pattern of first decline and then increase. Among them, the young group aged 15–24 had the highest incidence rate in 1990 but decreased the fastest (reaching the lowest point in 2000, p < 0.01), while the group aged 25–49 decreased more slowly (the lowest point occurred around 2010, p < 0.05), and all groups have shown an upward trend in recent years. It was especially significant in the 45–49 age group (p < 0.001); The change in prevalence rate is consistent with the trend of incidence rate. Mortality analysis showed that all age groups decreased significantly (p < 0.001). The decrease was the greatest from 1990 to 2000. The 15–19 age group remained at the lowest level all along. Although the 45–49 age group had the highest initial mortality rate (p < 0.001), the improvement was significant. After 2000, all age groups maintained a relatively low mortality rate (p < 0.01), but still showed gradient differences increasing with age. The changes in DALYs were consistent with the mortality trend (Figure S1).

### Average annual percent change

**Fig. 2:**
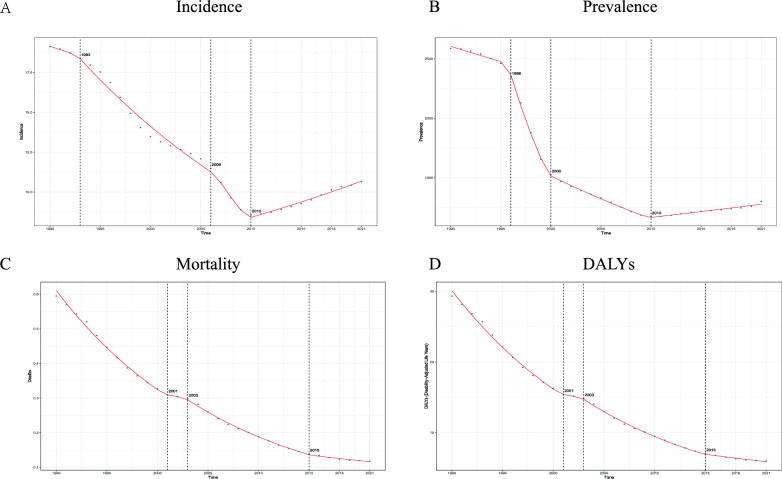
Temporal trends and average annual percentage change (AAPC) of chronic hepatitis C burden in China, 1990–2021. (**A**) Incidence; (**B**) Prevalence; (**C**) Mortality; (**D**) Disability-adjusted life years (DALYs). Red lines represent joinpoint-fitted trends, blue dots indicate observed annual values, and vertical dashed lines denote identified joinpoints that divide the study period into distinct segments for AAPC calculation

The analysis of the disease burden of chronic hepatitis C among women of reproductive age in China from 1990 to 2021 shows that the incidence rate presents a phased change characteristic. It decreased significantly from 1993 to 2006, the decline increased from 2006 to 2010, but rebounded after 2010. The prevalence rate also went through three stages: a rapid decline from 1996 to 2000, a steady decline from 2000 to 2010, and a recovery after 2010. Both DALYs and mortality rates have maintained a continuous downward trend. These results indicate that the prevention and control measures for chronic hepatitis C among women of reproductive age in China were highly effective from 1993 to 2010. However, the upward trend of the incidence and prevalence rates after 2010 suggests that the risk of new infections in this group needs to be vigilant (Fig 2).

Analysis of the epidemiological trend of acute hepatitis C among women of reproductive age in China from 1990 to 2021 shows that: Both the incidence rate and the prevalence rate showed a U-shaped change characteristic of first decreasing and then increasing. The changes were not significant in the initial stage (1990–1993), decreased significantly from 1993 to 2010, but turned to an upward trend after 2010. However, the mortality rate and DALYs maintained a continuous downward trend, among which the decline was the greatest from 2000 to 2007. Analysis of this group shows that the standardized incidence rate of acute hepatitis C rebounded after a decline from 1990 to 2010, and the mortality rate stabilized after a significant decline from 1990 to 2007. (Figure S2).

### Age-period cohort model

**Fig. 3:**
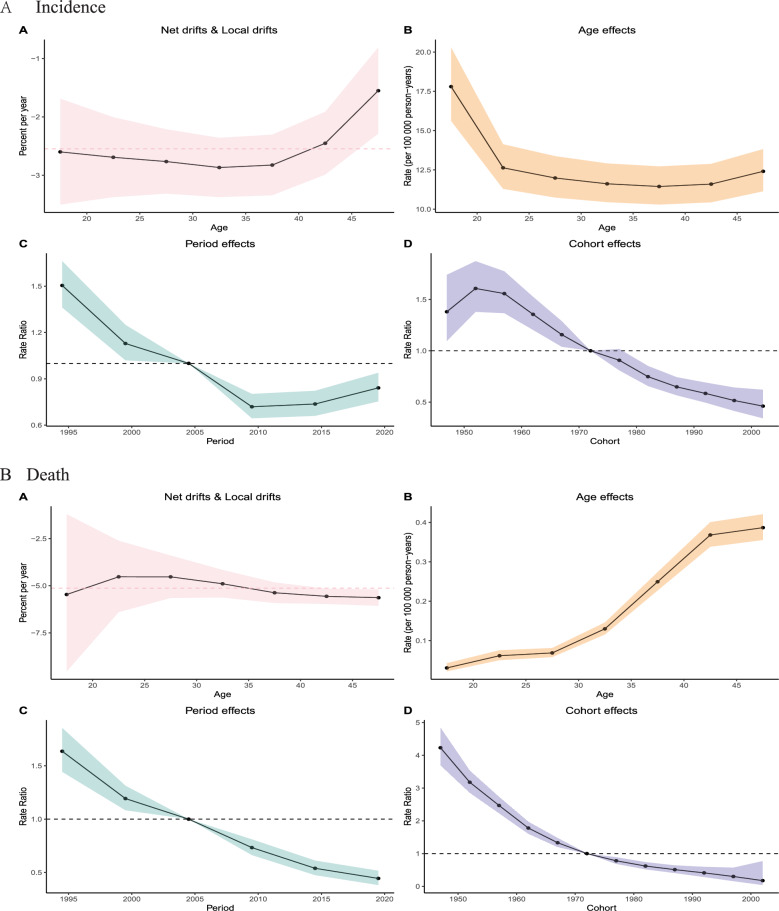
Age-period-cohort analysis of chronic hepatitis C incidence and mortality among Chinese women of reproductive age. (**A**) Net drift (pink dashed line) and local drifts, indicating overall and age‑specific annual percent changes, respectively. (**B**) Age effects: age‑specific incidence rates (per 100,000 person‑years) after adjustment for period and cohort effects. (**C**) Period effects: rate ratios relative to the reference period (2005, dashed line at 1.0). (**D**) Cohort effects: rate ratios relative to the reference cohort (1970, dashed line at 1.0). Shaded areas represent 95% confidence intervals

The age-period-cohort analysis of chronic hepatitis C among women of reproductive age in China from 1995 to 2021 revealed multi-dimensional epidemiological characteristics: The age effect shows that the incidence rate among those aged 20–45 decreases with age (from 20 per 100,000 to 12 per 100,000), but rebounds after the age of 45, while the mortality rate continues to increase with age (from 0.1 per 100,000 to 0.4 per 100,000). The period effect indicates that the incidence rate ratio rebounded after dropping from 1.5 in 1995 to 0.6 in 2015 (rising to 0.8 from 2015 to 2020), while the mortality rate ratio continued to improve (dropping from 1.75 to 0.5). The cohort effect shows that the morbidity and mortality rates of the birth cohort from 1950 to 2000 both present an intergenerational downward trend (the RR of morbidity decreased from 1.3 to 0.7, and the RR of mortality decreased from 5 to nearly 0). The net drift analysis showed that the annual change rate of the incidence rate across all age groups in this population was stable at -2% to -3%, and the mortality rate remained within the downward range of -7.5% to -2.5%. However, the improvement in the 40–45 age group was relatively weakened (Fig 3).

Age-period-cohort analysis of acute hepatitis C among women of reproductive age in China from 1995 to 2020 revealed multi-dimensional epidemiological characteristics: The age effect shows a U-shaped curve. The incidence rate among those aged 20–45 decreased from 30 per 100,000 to 20 per 100,000 and then rebounded to 25 per 100,000 at the age of 45. The mortality rate continued to decrease with age (0.06 per 100,000 at the age of 20 decreased to 0.00 per 100,000 at the age of 45). The period effect indicates that the incidence rate ratio has decreased from 1.5 to 0.6, and the mortality rate ratio has decreased from 7.5 to nearly 0. The cohort effect shows that the incidence rate of the birth cohort from 1950 to 2000 decreased from 1.5 to 0.5, and the mortality rate decreased exponentially (Figure S3).

### Bayesian age-period-cohort (BAPC) model for forecasting

**Fig. 4:**
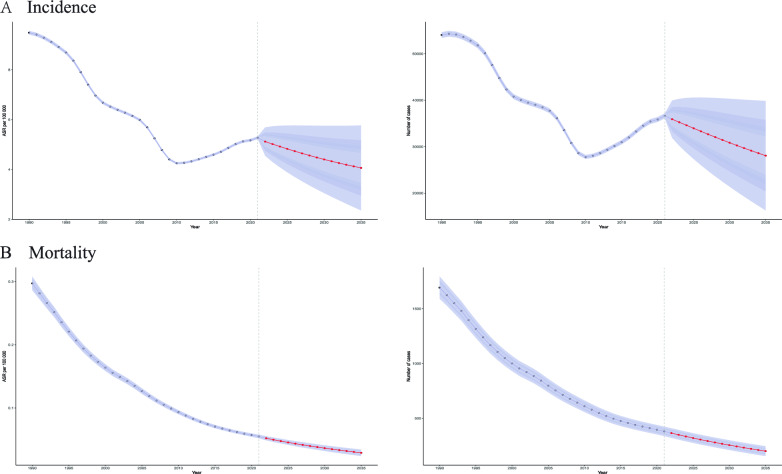
Bayesian age-period-cohort (BAPC) model projections of chronic hepatitis C incidence and mortality in China, 2021–2035. (**A**) Incidence projections: age‑standardized rate (ASR per 100,000 population, left axis) and estimated number of new cases (right axis). (**B**) Mortality projections: age‑standardized rate (ASR per 100,000 population, left axis) and estimated number of deaths (right axis). Gray solid lines with shaded bands represent historical observations (1990–2020) and corresponding 95% credible intervals; red dashed lines with purple bands represent predictions (2021–2035) and their 95% credible intervals. The vertical dotted line marks the start of the prediction period (2021)

Analysis of the epidemiological trend of chronic hepatitis C among women of reproductive age in China from 1990 to 2035 shows that: The age-standardized incidence rate (ASR) showed a fluctuating trend of first decreasing, then increasing, and then decreasing again. It decreased significantly from 1990 to 2005, briefly rebounded from 2005 to 2015, and then resumed to decline. During the same period, the number of cases decreased from 1990 to 2010 and rebounded from 2010 to 2020. The Bayesian age-period-cohort model predicts that ASR and the number of cases in this population will continue to decline between 2020 and 2035. Mortality analysis shows that both ASR and the number of deaths have continued to improve, significantly dropping from the peak in 1990 to the level in 2020. It is expected that they will further decrease in 2035, but the annual decline rate will gradually slow down (Fig. 4).

Analysis of the epidemiological trend of acute hepatitis C among women of reproductive age in China from 1990 to 2035 shows that both the age-standardized incidence rate (ASR) and the number of cases present dynamic changes of first decreasing, then increasing, and then decreasing again. ASR continuously decreased from the peak in 1990 to the lowest point in 2010 and then briefly rebounded. It is predicted that it will further decrease by 2035. Mortality analysis shows that ASR has stabilized after a significant decline between 1990 and 2010, and the changing trends of the number of deaths are similar. Based on the Bayesian age-period-cohort model, the projected age-standardized incidence and mortality rates for hepatitis C among women of reproductive age in China are expected to remain at historically low levels throughout the 2020–2035 period. The narrow prediction range indicates high credibility of the results. Analysis of this group shows that the incidence and mortality rates of acute and chronic hepatitis C have continued to decline from 2020 to 2030, and it is predicted that the mortality rate will approach zero by 2035 (Figure S4).

### Decomposition and analysis

Decomposition analysis of the hepatitis C disease burden among women of reproductive age in China and globally from 1990 to 2019 revealed contrasting trends. For chronic hepatitis C, the number of incident cases in this demographic in China decreased by 26,957, largely due to improvements in epidemiological factors (contribution: 101.66%), which offset the negative influence of population growth (− 57.16%). In contrast, incident cases globally increased by 156,332, primarily driven by population growth (101.19%) and aging (32.04%). A similar pattern was observed for mortality: China experienced a reduction of 1,075 deaths in this group, attributable to advances in epidemiological drivers (contribution: 147.48%), whereas global deaths rose by 6,021, mainly due to population growth (134.09%) and aging (93.04%).

**Fig. 5:**
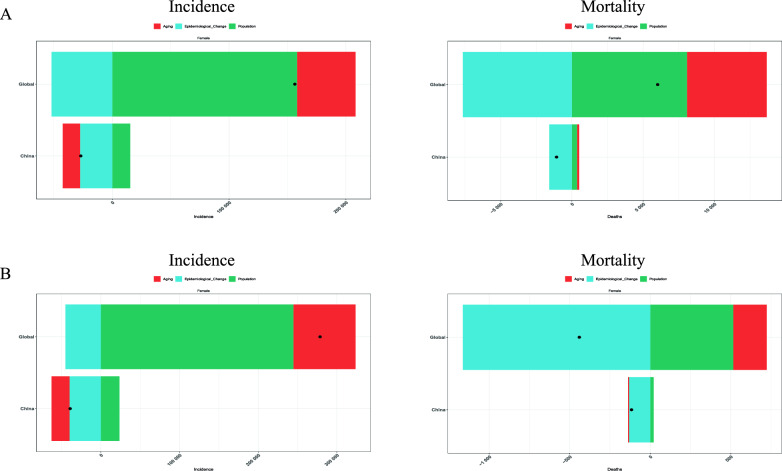
Decomposition Analysis of Hepatitis C Incidence and Mortality in China **A**: Chronic Hepatitis C **B**: Acute Hepatitis

For acute hepatitis C, China saw a substantial decline of 39,071 incident cases among women of reproductive age, resulting from improvements in epidemiological factors—including prevention and clinical management (contribution: 101.73%)—and a favorable contribution from population aging (58.61%), together outweighing the adverse effect of population growth (− 60.34%). Globally, acute hepatitis C incident cases increased by 278,645, predominantly due to population growth (87.81%) and aging (28.36%), despite a mitigating contribution from epidemiological improvements (− 16.17%) (Fig. 5).

## Discussion

The findings of this study highlight significant progress in hepatitis C virus (HCV) control among women of reproductive age in China from 1990 to 2021, as evidenced by the pronounced declines in age-standardized incidence, prevalence, mortality, and DALY rates, all of which surpassed global averages. The sustained decline in the incidence of acute and chronic hepatitis C in this population, as indicated by estimated annual percentage changes (EAPCs) of –2.512% and –2.557% respectively,reflects the effectiveness of China’s public health interventions for this group, including enhanced screening, antiviral therapy scaling, and harm reduction strategies. Despite these achievements, China remains a high-burden country for this demographic, accounting for 12.5% of global chronic HCV cases among women of reproductive age in 2021, underscoring the need for sustained efforts to eliminate HCV [28]. The remarkably low mortality (ASDR = 0.118/100,000) and DALY rates (6.005/100,000) in this group compared to global figures suggest that China’s healthcare system has successfully mitigated severe HCV outcomes for women of reproductive age, likely due to improved access to direct-acting antivirals (DAAs) and better management of cirrhosis and hepatocellular carcinoma [29, 30]. However, the persistence of 4.235 million chronic HCV patients among women of reproductive age in China indicates ongoing transmission and diagnostic gaps within this group, necessitating targeted strategies such as high-risk population screening, public awareness campaigns, and equitable treatment access [31]. The accelerated decline in HCV burden among women of reproductive age in China demonstrates the potential for China to achieve WHO elimination targets for this group, but continued investment in prevention, diagnosis, and treatment is critical to address remaining challenges [32].

Average annual percent change reveals dynamic epidemiological shifts in hepatitis C burden among women of reproductive age in China from 1990 to 2021, with distinct inflection points reflecting the impact of public health interventions and emerging challenges. The marked decline in chronic hepatitis C incidence and prevalence in this population from 1993 to 2010 likely stems from improved blood safety measures and early antiviral treatments, while the post-2010 rebound suggests either surveillance intensification, risk behavior resurgence, or limitations in sustaining prevention coverage within this group. The infection rate of acute hepatitis C among women of reproductive age in China shows a fluctuating trend of first decreasing and then increasing. This phenomenon is particularly prominent in this group [33]. Epidemiological monitoring data indicate that the infection rate of this group has significantly increased since 2010, while the disease burden indicators (including mortality rate and disability-adjusted life years) for this population have maintained a continuous improving trend during the same period [34]. This deviation between the infection rate and the disease burden indicators may reflect the current situation where the improvement in diagnostic capabilities is not synchronized with the implementation of prevention and control measures for women of reproductive age, suggesting the need to formulate more precise intervention strategies for high-risk populations within this group [35]. The persistent mortality reduction across all phases underscores the life-saving role of advancing HCV therapeutics for women of reproductive age, yet the resurgence in transmission signals the need for reinvigorated harm reduction strategies, especially among high-risk populations within this group [36]. These findings collectively emphasize that while China’s historical interventions for women of reproductive age achieved remarkable success, the evolving epidemiology demands adaptive strategies combining targeted screening, treatment-as-prevention approaches, and renewed focus on transmission hotspots to counteract recent upward trends in this population.

The age-period-cohort (APC) analysis provides critical insights into the evolving epidemiology of hepatitis C among women of reproductive age in China, revealing distinct age-specific risks, period-driven fluctuations, and generational improvements in disease burden. The rebound in chronic hepatitis C incidence after age 45 in this population, coupled with steadily increasing mortality in older populations, suggests late-age infection persistence or reactivation, possibly due to historical exposure risks or inadequate screening in middle-aged and elderly cohorts of women of reproductive age. The period effect, characterized by an incidence rebound post-2015 despite sustained mortality declines in this group, aligns with Average Annual Percent Change findings and likely reflects gaps in prevention or delayed treatment access during this phase, even as therapeutic advances (e.g., DAA rollout) drove mortality reductions for this population [37]. Significantly, the intergenerational decline in both morbidity and mortality across birth cohorts (1950–2000) among women of reproductive age in China underscores the cumulative benefits of public health measures, including blood safety reforms and harm reduction programs, though the attenuated improvement in the 40–45 age group signals a need for targeted interventions in this demographic within this population [38, 39]. For acute hepatitis C, the U-shaped age-specific incidence—with elevated risks in younger and older adults—may indicate behavioral risks (e.g., injection drug use) in youth and missed diagnoses or immune senescence in older populations among women of reproductive age [40], while the exponential mortality decline across cohorts highlights China’s success in averting severe outcomes for this group [41]. Together, these APC findings emphasize the importance of age-tailored screening, particularly for older adults with chronic HCV and younger populations at risk of acute infection among women of reproductive age, alongside period-specific strategies to address emerging transmission dynamics and sustain generational gains toward elimination in this population.

The Bayesian Age-Period-Cohort (BAPC) projections for hepatitis C among women of reproductive age in China from 1990 to 2035 demonstrate a complex but ultimately optimistic trajectory, with key inflection points reflecting both historical challenges and future opportunities for elimination in this group [42]. The model predicts a sustained decline in age-standardized incidence (ASR) and mortality for both chronic and acute hepatitis C among women of reproductive age in China through 2035, building upon the substantial reductions achieved since 1990—particularly in mortality, which is projected to approach zero among women of reproductive age by 2035 [43]. However, the transient rebounds in incidence between 2005–2015 (chronic HCV) and post-2010 (acute HCV) in this population underscore lingering vulnerabilities in prevention, possibly tied to gaps in high-risk population interventions or delayed treatment scale-up. The anticipated slowdown in mortality decline rates suggests that residual burdens may persist among older cohorts with advanced liver disease within this group, emphasizing the need for enhanced fibrosis screening and timely DAA access for women of reproductive age [44, 45]. Significantly, the narrow confidence intervals in acute HCV projections reinforce the reliability of these trends for this population, likely reflecting China’s robust harm reduction policies for women of reproductive age. These forecasts highlight that while China is on track to meet WHO elimination targets for women of reproductive age, maintaining momentum will require addressing the drivers of past incidence rebounds—such as targeted testing in older populations born before widespread blood safety reforms—and ensuring equitable treatment access to avert future resurgences in this group [46]. The convergence of declining incidence and mortality signals a promising transition from HCV control to elimination among women of reproductive age in China, provided current interventions are sustained and adapted to emerging epidemiological shifts within this population.

The decomposition analysis underscores China’s remarkable success in hepatitis C control among women of reproductive age compared to global trends, demonstrating how targeted public health strategies can counteract demographic pressures. While many countries—particularly low- and middle-income nations—face rising HCV burdens due to population growth and aging (accounting for > 200% of global incidence increases), between 1990 and 2019, China achieved a reduction of 26,957 chronic and 39,071 acute hepatitis C cases among women of reproductive age, gains primarily attributable to epidemiological improvements—including prevention scaling and DAA access—which contributed over 100% to the decline [47]. This divergence highlights that China’s investments in blood safety, harm reduction, and treatment infrastructure for this group not only neutralized demographic challenges (-57% impact from population growth) but also generated net declines, whereas global mortality rose by 6,021 cases due to unmitigated aging effects. The findings emphasize two critical policy insights: first, China’s model for women of reproductive age proves that rapid DAA scale-up and prevention can decouple disease burden from demographic trends [48]; second, the persistent global increases signal that elimination efforts must prioritize regions with youthful populations (requiring enhanced prevention) and aging cohorts (needing expanded treatment access) [49]. Significantly, China’s 58.6% contribution from aging to acute HCV reductions among women of reproductive age suggests its interventions effectively protected older adults within this group—a lesson for countries where aging drives rising burdens [50]. These results advocate for differentiated strategies: population-dense regions require amplified primary prevention, while aging societies need integrated screening/treatment programs to replicate China’s success among women of reproductive age in turning demographic headwinds into epidemiological gains.

From 1990 to 2021, the disease burden indicators related to acute and chronic hepatitis C-induced liver diseases among women of reproductive age in China showed a general downward trend. However, due to the large population base, a large number of current infected individuals within this group, and still relatively low diagnosis rates and treatment coverage rates, China still faces significant challenges in achieving the World Health Organization’s goal of "eliminating the public health threat of HCV by 2030" for women of reproductive age. Currently, effective measures need to be taken to improve the accessibility of diagnosis and treatment for hepatitis C-related liver diseases (especially HCV-induced liver cancer) for women of reproductive age, and optimize the diagnosis and treatment model to reduce the economic burden on patients in this group. Future research should focus on exploring the risk factors for death from hepatitis C-related liver diseases among women of reproductive age in China, providing scientific basis for formulating precise prevention and control strategies for this population. The advancement of this research direction will play an important role in promoting the achievement of the HCV elimination goal for women of reproductive age in China.

Several limitations should be considered when interpreting these findings. First, the estimates rely on modeled data from the Global Burden of Disease study, which may be subject to inaccuracies due to underreporting or regional disparities in surveillance. Second, while age-period-cohort and decomposition analyses revealed multidimensional epidemiological features for women of reproductive age in China, residual confounding and ecological bias cannot be fully ruled out. Third, although Bayesian prediction models showed narrow uncertainty intervals, long-term forecasts remain speculative, particularly regarding future behavioral and policy shifts specific to this population. Finally, the study focused primarily on women of reproductive age, potentially overlooking other high-risk groups, and the results may not fully capture recent changes in diagnosis and treatment practices after 2021 for this group.

## Conclusion

Based on the analysis of the burden of hepatitis C virus (HCV) diseases among women of reproductive age in China from 1990 to 2021, this study shows that the age-standardized incidence and prevalence of acute and chronic HCV in this population have shown a significant downward trend, with the decline rate exceeding the global average; in 2021, the number of chronic HCV cases among women of reproductive age in China accounted for 12.5% of the global total, while the mortality and disability-adjusted life year (DALY) rates were significantly lower than the global level and showed a more rapid decline. The epidemiological trend of women of reproductive age presented a stratified age characteristic: the incidence of chronic HCV decreased first and then increased among different age groups, while the prevalence generally decreased; the incidence of acute HCV showed a U-shaped trajectory, and the mortality rate decreased significantly. Trend analysis revealed that the incidence and prevalence of chronic and acute HCV among women of reproductive age in China respectively reached a turning point around 2010, while the mortality rate and DALY rate continued to decline throughout the study period. The age-period-cohort model further revealed that the incidence of chronic HCV among women of reproductive age in China rebounded after the age of 45, and after 2015, it showed a risk rebound driven by the period effect, but the birth cohort effect indicated that the incidence and mortality risks of subsequent cohorts gradually decreased; acute HCV presented an U-shaped age curve for incidence, and the period and cohort effects supported the continuous decline in its incidence and mortality in this population. Bayesian prediction models indicated that by 2035, the age-standardized incidence and case numbers of acute and chronic HCV among women of reproductive age in China are expected to continue to decline, and the mortality rate will remain at a historical low. Decomposition analysis pointed out that the significant reduction in HCV incidence among women of reproductive age in China was mainly attributed to the positive contribution of epidemiological progress, which offset the pressure brought by population growth, which is in sharp contrast to the pattern of increased incidence worldwide, which is mainly driven by population growth and aging.

## Supplementary Information

Below is the link to the electronic supplementary material.

Supplementary figure 1.

Supplementary figure 2.

Supplementary figure 3.

Supplementary figure 4.

## Data Availability

The data used in this study can be derived from the GBD 2021 (Available at: https://ghdx.healthdata.org/gbd-2021).

## Abbreviations

HCV: Hepatitis C Virus
ASR: Age-Standardized Rate
DALY: Disability-Adjusted Life Year
APC: Age-Period-Cohort
EAPC: Estimated Annual Percentage Change
GBD: Global Burden of Disease
IHME: Institute for Health Metrics and Evaluation
BAPC: Bayesian Age-Period-Cohort
ASIR: Age-Standardized Incidence Rate
ASPR: Age-Standardized Prevalence Rate
ASDR: Age-Standardized Death Rate (or Mortality Rate)
UI: Uncertainty Interval
CI: Confidence Interval
DAA: Direct-Acting Antiviral
WHO: World Health Organization
LMICs: Low- and Middle-Income Countries
NNDRS: National Notifiable Disease Reporting System
RR: Rate Ratio
HCC: Hepatocellular Carcinoma
