# Supplementary figures and images for: The three-decade trajectory of hepatitis C burden among women of reproductive age in China: a retrospective and predictive study
Source: Virol J. 2026 May 21;23:127. doi: 10.1186/s12985-026-03079-4 (PMC13191844; doi:10.1186/s12985-026-03079-4)

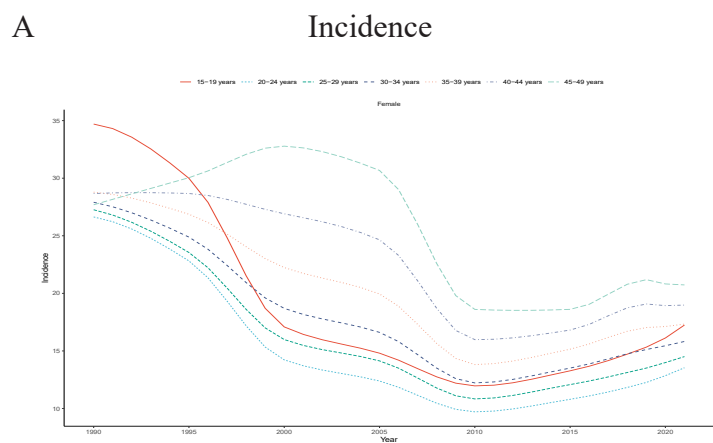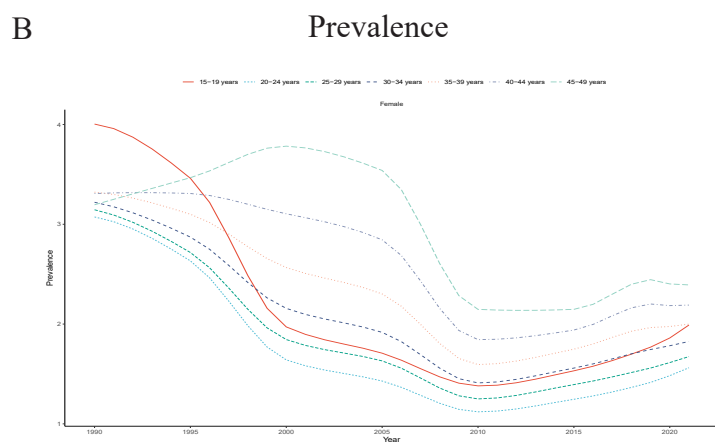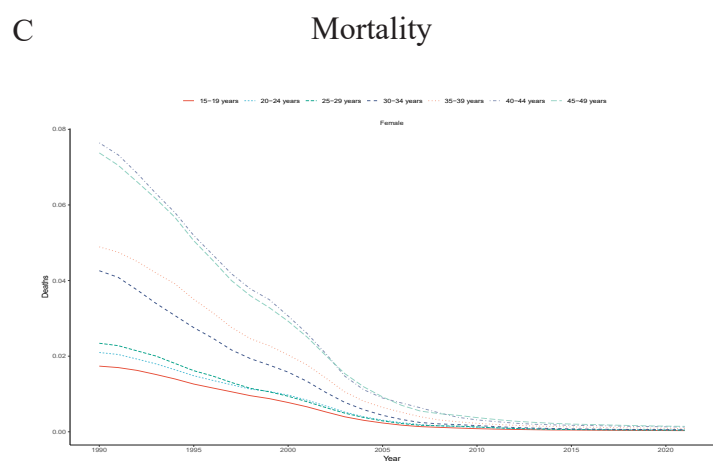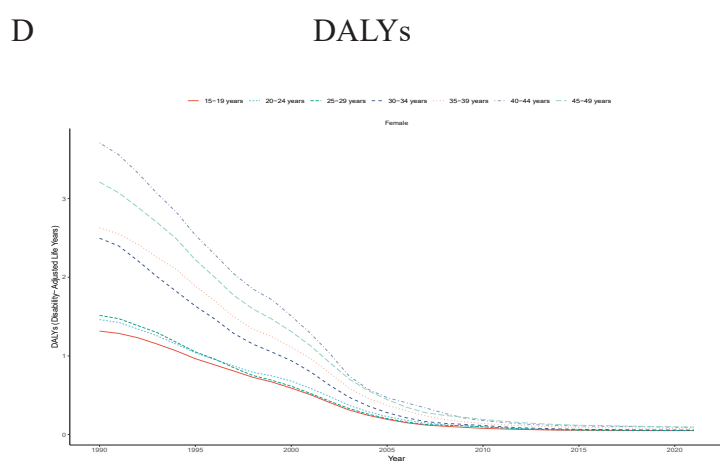

Figure S1. Trends in Acute hepatitis C Incidence, Prevalence, Mortality, and DALYs by Age

Supplement: Supplementary file 1 — Supplementary figure 1. [file 12985_2026_3079_MOESM1_ESM.pdf]
