# Supplementary material for: The three-decade trajectory of hepatitis C burden among women of reproductive age in China: a retrospective and predictive study
Source: Virol J. 2026 May 21;23:127. doi: 10.1186/s12985-026-03079-4 (PMC13191844; doi:10.1186/s12985-026-03079-4)
Supplement: Supplementary file 2 — Supplementary figure 2. [file 12985_2026_3079_MOESM2_ESM.pdf]

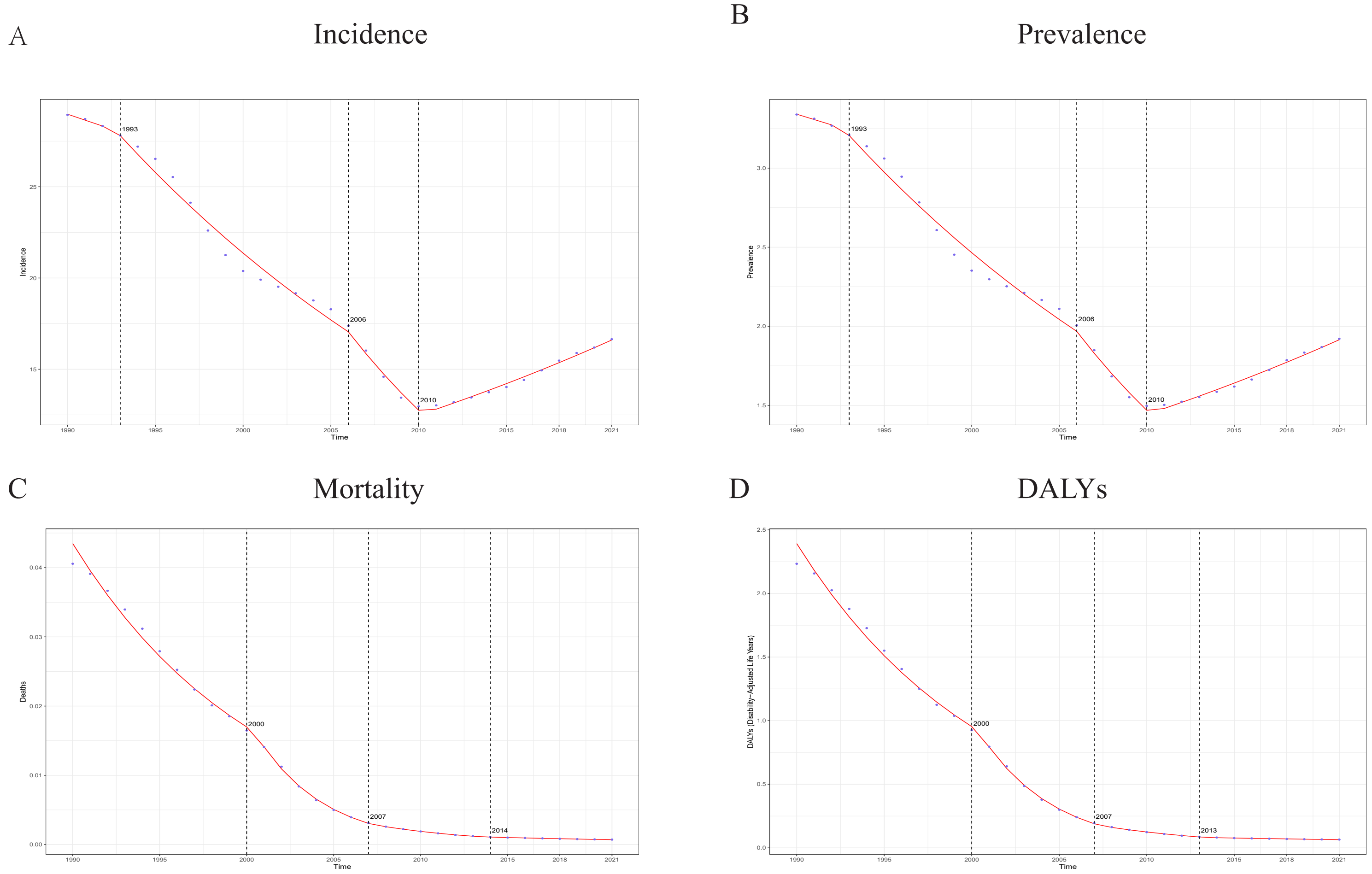

Figure S2. Annual Average Percentage Change (AAPC) in Acute hepatitis C Incidence, Prevalence, Mortality, and DALYs (1990-2021)， In all panels, the red line indicates the overall trend, and the vertical dashed lines highlight years where there were notable changes in the trend. The data points are marked with blue crosses.
