# Supplementary material for: The three-decade trajectory of hepatitis C burden among women of reproductive age in China: a retrospective and predictive study
Source: Virol J. 2026 May 21;23:127. doi: 10.1186/s12985-026-03079-4 (PMC13191844; doi:10.1186/s12985-026-03079-4)
Supplement: Supplementary file 3 — Supplementary figure 3. [file 12985_2026_3079_MOESM3_ESM.pdf]

A Incidence

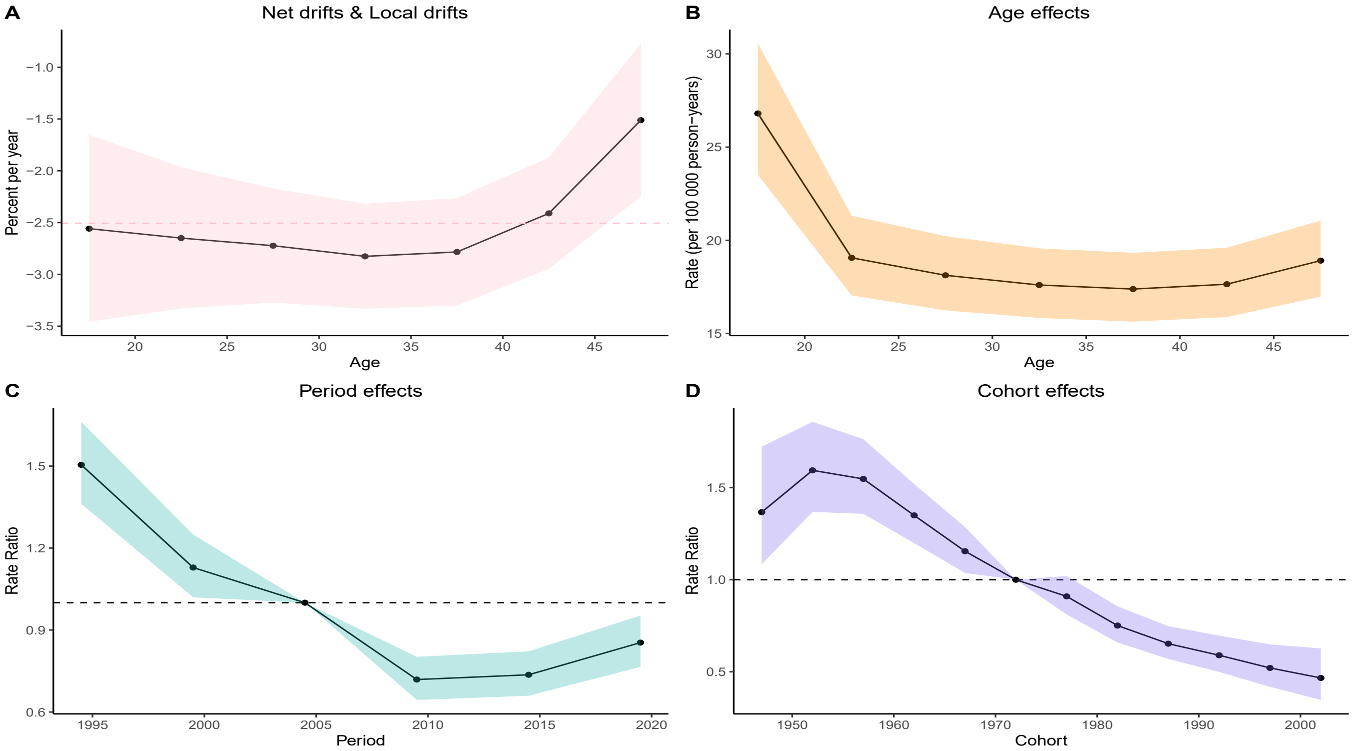

B Death

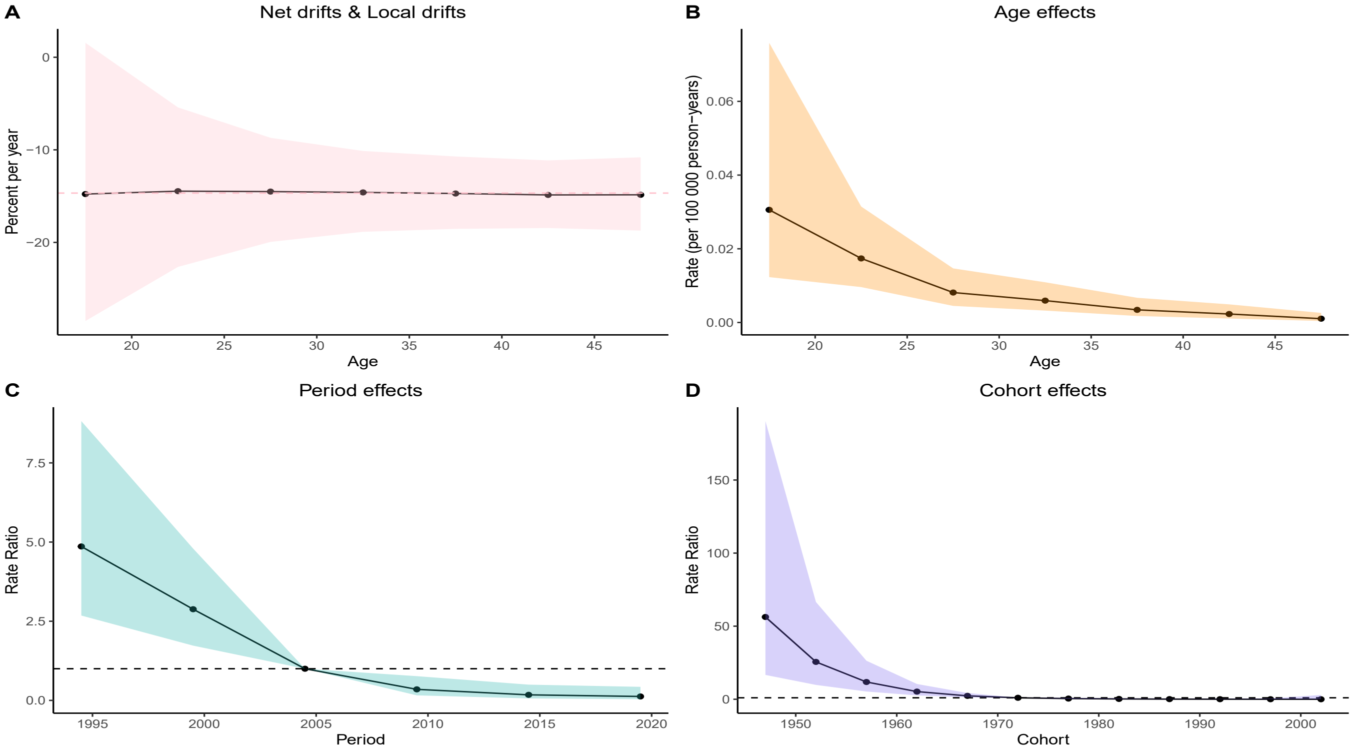

Figure S3. Age-Period-Cohort (APC) Analysis of Acute hepatitis C Incidence and Death
