# Supplementary material for: The three-decade trajectory of hepatitis C burden among women of reproductive age in China: a retrospective and predictive study
Source: Virol J. 2026 May 21;23:127. doi: 10.1186/s12985-026-03079-4 (PMC13191844; doi:10.1186/s12985-026-03079-4)
Supplement: Supplementary file 4 — Supplementary figure 4. [file 12985_2026_3079_MOESM4_ESM.pdf]

A Incidence

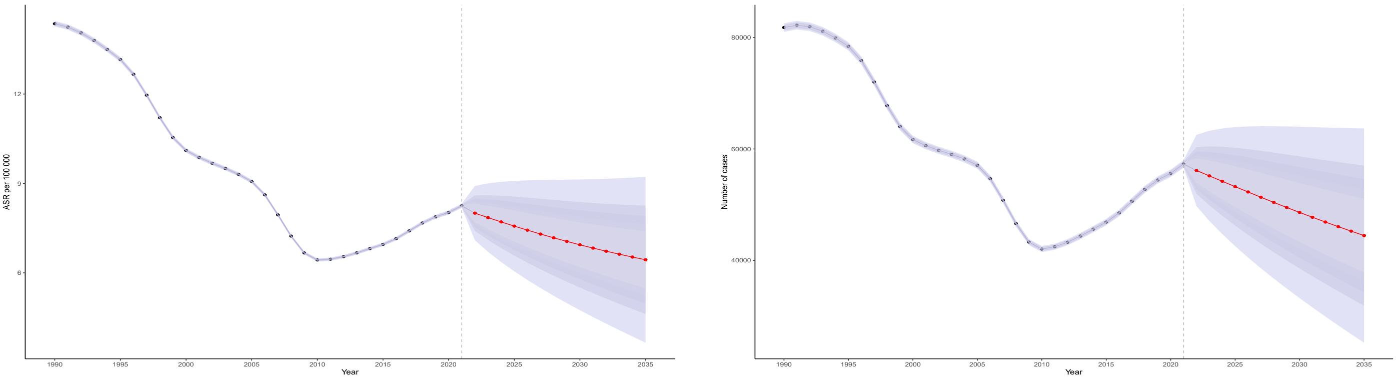

B Mortality

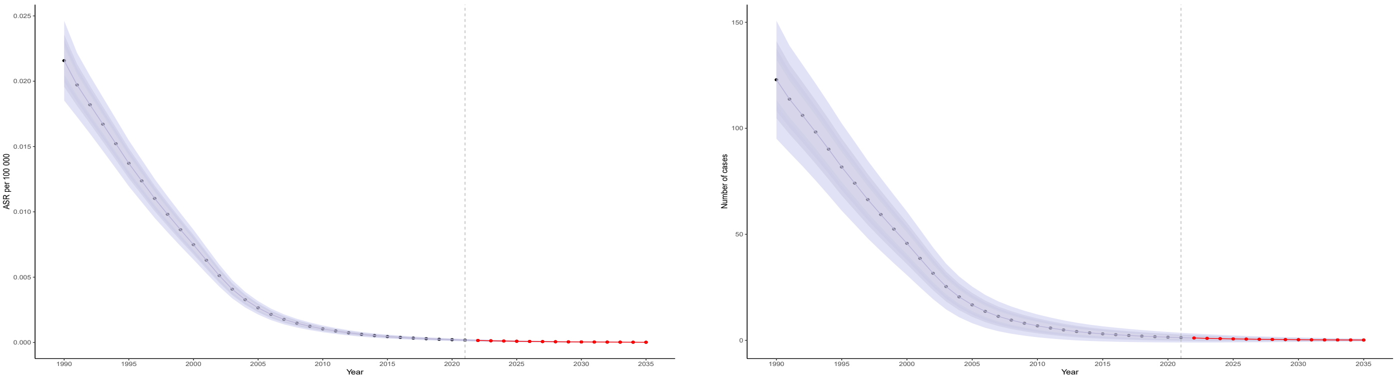

Figure S4. Bayesian Age-Period-Cohort (BAPC) Model Predictions for Incidence and Mortality of Acute hepatitis C
